# Busman’s stomach and the embodiment of modernity

**DOI:** 10.1080/13619462.2016.1226806

**Published:** 2016-09-26

**Authors:** Rhodri Hayward

**Affiliations:** ^a^ Centre for the History of the Emotions, School of History, Queen Mary University of London, London, UK

**Keywords:** Modernity, trade unions, transport, temporality, digestive disorders

## Abstract

This paper examines the relationship between the gastric illness, ‘busman’s stomach’ and the Coronation bus strike of May 1937 in which 27,000 London busworkers walked out for better working conditions and a seven-and-half-hour day. It explores the way in which new patterns of somatisation, gastroenterological techniques, psychological theories and competing understandings of time worked together to create new political institutions and new forms of political action in inter-war Britain.

I sometimes wonder … what kind of animal you are going to produce in the end, with all the strap-hanging in buses and tubes, what with balancing tricks and all the crushing. I wonder what the future man and woman will be like

Ernest Bevin, *Evidence to the Industrial Court of Enquiry*, 3 May 1937, 21

Golfer’s stomach, bus driver’s stomach! What poor things we are!

James Lansdale Hodson, *Home Front* (London: Victor Gollancz, 1942), 181

This is an essay about gastritis and the ways that stomach pains, sickness and diarrhoea can help create new political institutions and make possible new kinds of political action. It focuses on the history of ‘busman’s stomach’—a stress-related disorder that rose to prominence during the inter-war years—and its relationship to the Coronation Bus Strike—a four-week walkout by 27,000 London bus drivers and conductors in May 1937.^1^ Today, the bus strike is largely remembered for the tensions it revealed between the rank and file workers movement (an unofficial network of local workers’ committees part-led, initially, by the Communist Party of Great Britain) and the executive of the Transport and General Workers Union (TGWU) under its general secretary, Ernest Bevin.^2^ Indeed, among left-wing commentators, the strike is seen as marking the betrayal of one of the first tentative experiments in British syndicalism. As Bill Jones, one of the busmen’s leaders later reminisced, the ‘executive committee [of the TGWU] had never any intention of assisting the busmen; and instead were “all the time concerned about destroying the rank and file movement”’.^3^ This complaint has taken firm root in the radical imagination. Writing in *International Socialism* in 1976, Pete Glatter argued that Bevin exploited the strike to ‘play the busmen like a cat with its prey’.^4^ A decade later, the TGWU shop steward, Ken Fuller would rehearse the claim that the strike was prolonged through the tacit agreement of Bevin and Lord Ashfield, the chairman of the London Passenger Transport Board in order to exhaust, isolate and undermine the Rank and File Movement.^5^ Despite recent efforts by historians to downplay the idea of conflict between union leaders and the rank and file in British labour history, in the busmen’s case, that opposition has been acknowledged by both sides.^6^

The pessimism of these analyses is perhaps compounded by the failure of left-wing commentators to recognise the crucial mediating role of busman’s stomach in this dispute. The illness was central to the union’s case and, as I want to make clear, part of the radicalism of the busmen’s claim lay in their ability to turn a dispute over working practices into a clinical debate over the aetiology of a disease. Although the strike may have failed to realise its initial aims and, indeed, did lead to the fracturing of the busmen’s rank and file, it also encouraged a more radical transformation. It inaugurated a shift in the conduct of labour relations: away from competing claims based upon ideas of custom, rights and duty to a new kind of dispute conducted through reference to theories of psychophysiology and endocrinology. It can be seen as a movement from a politics based upon ‘moral economy’—a politics in which, as E. P. Thompson argued, workers’ rights were established through reference to custom and historical memory—to what we can call a ‘psychological economy’ in which theories of the body and its physical and psychological capacities play a crucial role in mediating and articulating the worker’s demands.^7^

The emergence of this new rhetoric can be traced back to the early twentieth century, when continental writers drew upon new laboratory studies of fatigue to argue for a ‘physiological limit’ to the working day.^8^ By the early 1930s, this physiological argument had been supplemented by a more holistic approach that emphasised the effects of labour on the worker’s body and personality. The appearance of this new kind of political contestation was not a straightforward achievement. It rested upon a heterogeneous collection of innovations ranging from new procedures in insurance administration and epidemiological investigation through to the development of novel clinical techniques, diagnostic technologies and new patterns of somatisation. More specifically, it was sustained by the production of alternative models of temporality. As we shall see, the busmen’s case, and the idea of the psychological economy that it was founded on rested upon the juxtaposition of different forms of time: the lived time of the body; the pressured time of modernity; and the evolutionary time of prehistoric environment. It was the radical disjuncture between these forms of time that made the new politics possible.

## Modernity and the making of magical objects

Modernity has attracted detailed attention from historians.^9^ They tend to depict it as a corrosive force. Drawing upon a mixture of Marx and Weber, historians claim that processes of state rationalisation and the increasing pace of production and consumption work together to bring about the breakdown of traditional values and institutions. Marx and Engels’s poetic description of a condition of relentless instability in which ‘all fixed fast frozen relations . . . are swept away and new ones become antiquated before they can ossify’ has served as the basis for a broad range of analyses that describe the gradual disenchantment of the world and the sublimation of custom and convention—the foundations of Thompson’s moral economy—into the ‘iron cage’ of bureaucratic rationality.^10^ As Weber explained, in place of a commonwealth of values rooted in ‘a religious ethic of brotherliness’, there emerged new schemes in which every aspect of life was opened up to the promise of instrumental calculation. Turning to the modern experience of public transport, Weber argued that it was this promise of instrumental calculation that brought about the disenchantment of the world.

Does it mean that we, today, for instance, everyone sitting in this hall, have a greater knowledge of the conditions of life under which we exist than has an American Indian or a Hottentot? Hardly. Unless he is a physicist, one who rides on the streetcar has no idea how the car happened to get into motion. And he does not need to know. He is satisfied that he may 'count' on the behavior of the streetcar, and he orients his conduct according to this expectation; but he knows nothing about what it takes to produce such a car so that it can move. The savage knows incomparably more about his tools …. The increasing intellectualization and rationalization do not, therefore, indicate an increased and general knowledge of the conditions under which one lives. It means something else, namely, the knowledge or belief that if one but wished, one *could* learn it at any time. Hence, it means that principally there are no mysterious incalculable forces that come into play, but rather that one can, in principle, master all things by calculation. This means that the world is disenchanted. One need no longer have recourse to magical means in order to master or implore the spirits, as did the savage, for whom such mysterious powers existed. Technical means and calculations perform the service.

^11^Yet, this process of disenchantment could never be completed. As Marx noted as early as 1856, ‘In our days, everything seems pregnant with its contrary’.^12^ Just at that moment when capitalist modernity was banishing magic from the world, it was also bringing forth new kinds of monstrous forces that animated dead materials and bewitched living beings. Part of this process, the fetishisation of commodities and the creation of an enchanted world of consumer goods, has attracted detailed attention from historians inspired by the writings of Walter Benjamin. And this analysis has been extended beyond the world of consumption in the work of Michael Saler, Alex Owen and Jane Bennett, who depict modernity as a form of re-enchantment that imbues the world with new values, unleashes alternative temporalities and creates new spaces for the imagination.^13^ This essay extends their claim. However, whereas Saler and others have largely explored this process through an examination of literary experiments and stage magic, I argue that the work of re-enchantment is driven by much more prosaic processes.

As recent work in the sociology of science has shown, new objects (such as revolutionary movements and gastric illnesses) emerge in our world through an ongoing traffic between nature and culture. Our representations of nature take on a life of their own as they link up with new materials: stockholder certificates, membership lists or barium meals. Modernity, as Bruno Latour has argued, is populated with hybrid creatures produced through an ongoing traffic across a network of elements.^14^ As such networks grow, as a truth claim moves from being a simple assertion of correspondence between an individual’s words to and the world, to involve reference to a wider web of supporting phenomena (such as footnotes, lobby groups and sickness returns), such claims take on an increasing objectivity. The labour and assertion that lie behind such claims is obscured (or ‘blackboxed’ to use Latour’s phrase) behind the overgrowth of linked objects and interests that now surround them. The forces of bureaucratic rationalisation did not simply disenchant the world as Weber had insisted; rather, as the story of busman’s stomach demonstrates, it was the compression of time and space and the avalanche of techniques and materials that accompanied this that allowed the disenchanted world to be repopulated with the magical and monstrous hybrids that Marx and Latour describe.^15^

It was in the struggle over times and schedules that the Rank and File Movement (RFM) was born. At the end of July 1932, the London General Omnibus Company, the largest of London’s bus companies, announced that staff wages would be reduced and 800 jobs lost over the following month due to the decline in receipts during the recession.^16^ In response to this threat, Bevin on behalf of the TGWU brokered a settlement in which the posts were saved and the pay reduction tempered, in return for his members accepting tighter schedules. The new schedules entailed a shift in the average driving speed from 9.3 to 14.3 miles per hour—a shift that necessitated a speed of around 30mph between stops.^17^ The agreement was widely criticised on the shop floor. On 12 August, a meeting of delegates from the London garages, chaired by Bert Papworth, secretary of the Chelverton Branch, attacked the supine attitude of Bevin and the Central Bus Committee.^18^ Eighteen days later, members picketed Transport House (the headquarters of the TGWU) and briefly occupied the building. Initially, Bevin blamed this militancy on Communist agitators and it was true that the party had been engaged in an ongoing attempt to wrest control of British trade unionism.^19^ However, by 1933, it had only managed to recruit a dozen members among the busworkers.^20^ The campaign instead was largely autonomous—coordinated through local garage committees and given voice through a cyclostyled news-sheet, *Busman’s Punch*—which provided a forum for dissent against both employer and union. As Ken Fuller has noted, the movement was sustained in part by the workplace structure of the bus garages, in which large groups of men were able to congregate during downtime for encouragement and discussion, and in part by the complex hierarchy of representative committees within the Passenger Transport Section of the TGWU.^21^ Garages elected their own branch secretaries, depot representatives and conference delegates, who in turn reported to area conferences, while an autonomous Central Bus Committee within the union coordinated demands.^22^ Moreover, under the Anderton’s Hotel Agreement, which had led to the incorporation of the busmen’s union (the Union of Vehicle Workers) into the TGWU, the Central Bus Committee was granted its own full-time secretary and given direct access to the union executive.^23^ Alongside the traditional union hierarchy, the RFM established a new network, with six delegates from each garage meeting monthly and in turn electing organising and editorial committees.^24^ The RFM was chaired by Frank Snelling with Bernard Sharkey serving as his deputy and Bert Papworth as official organiser.

The movement exasperated the union. On 17 January 1933, a wild cat strike broke out in response to the posting of new, speeded up schedules at Forest Gate Bus Garage. Five hundred busmen walked out with 300 participating in a picket.^25^ Within four days, another 26 garages had stopped work with another 13,000 drivers and conductors joining the strike.^26^ Six months later at the biennial TGWU conference, Bevin resurrected his claim that the RFM was a front for the Communist Party of Great Britain. The movement’s activists responded by taking five out of six seats on the Central Bus Committee and working towards the creation of the TOT—the Trains, Omnibus and Tramworkers organisation which would allow for coordinated action across the transport industry. In the case of the busmen at least, the move towards speed and rationalisation did not break down communal values but instead led to the emergence of new radical institutions.^27^

The London Passenger Transport Board, the second actor in this narrative, was also born of the movement towards rationalisation. The financial pressures that had been used to justify the 1932 ‘Speed Up’ agreement also led to a period of centralisation and consolidation in the provision of metropolitan transport services. Labour MPs had long decried the levels of waste and duplication in metropolitan transport services: wastage caused by unfettered competition between private companies.^28^ In June 1933, through the efforts of Herbert Morrison, the London Passenger Transport Board was established. It amalgamated 5 underground railways, 17 tramways, 136 bus and coach undertakings and 4 subsidiary companies.^29^ The new company was led by Lord Ashfield, the director of the London Underground Electric Railway and former President of the Board of Trade.^30^ He was aided by his old deputy, Frank Pick, who became vice chairman.^31^

Ashfield and Pick shared a faith in the possibility of planning and the promise of modernity. Both men imagined their commercial mission in cosmological and evolutionary terms. Shortly before his appointment to the LPTB, Pick gave a presentation to West Country designers warning that ‘fluidity, plasticity [*sic*] are the sovereign virtues for survival’ and that those ‘races and genera’ which failed to adapt were condemned to extinction.^32^ Pick returned to this organicist theme in 1938 Studd Lecture on Administration and the Individual, arguing that all corporations ‘must decay and eventually die’ unless they can rejuvenate through constant reorganisation.^33^ Pick rationalised his celebration of speed and organisation through reference to the work of the popular physicist, Arthur Eddington.^34^ The problem of cosmic entropy could only be combated through commitment to relentless dynamism. It was a commitment that would require some sacrifice on the part of the workers.^35^ As Pick explained to LPTB staff: ‘The management of a great undertaking must be in continual warfare with an invisible enemy … the mischievous imps of waste’. Yet, a ‘trivial increase’ in efficiency of just 3 per cent would, Pick thought, be enough to set the Board on the path to ‘Utopian bliss’.^36^ This ongoing contest between the Maxwellian demon of managerial efficiency and the ‘imps of waste’ was played out in the organisation’s internal struggles over speed and temporality. It was in these struggles that busman’s stomach first emerged.

Busman’s stomach was a transient condition.^37^ Cases were first highlighted at the trade union-funded Manor House Hospital in the late 1920s and by the early 1930s gastric disorder was widely understood to be a hazard of modern buswork.^38^ Busmen interviewed for Hubert Llewelyn Smith’s *New Survey of London* (1933–1934) attributed the new illness to their hurried and irregular consumption of picnic lunches, the sedentary work of driving, carbon monoxide fumes and the unending pressure experienced in their work*.* ^39^ This commonplace connection of the busman’s plight to the problems of modernity drew upon a long-standing argument in British medicine that linked emotional strain to alimentary health. In the writings of eighteenth- and early nineteenth-century physicians such as George Cheyne and James Johnston, morbid digestion was associated with national epidemics of hypochondria and nervous debility: epidemics driven by the pursuit of luxury and the demands of commerce.^40^ Yet, although the association between mental distress, modernity and gastric disorder was long-standing, the basis of this connection had been understood in very different ways. In eighteenth- and nineteenth-century writings, it was morbid digestion that disrupted mind and character. The stomach developed tics and idiosyncrasies in response to bad meals and overeating, which in turn lowered the individual’s mood or robbed them of nervous energy.^41^ In the twentieth century, however, this understanding of the relationship between mind and stomach was reversed. Digestive disturbances were no longer seen as the cause of psychological complaints but instead were seen as their outcome. Stress undermined digestion. It disrupted peristalsis causing digestive enzymes and hydrochloric acid to accumulate in the duodenum and stomach, respectively. This accumulation in turn generated a range of illnesses from gastritis to ulceration. Psychological equilibrium was now intimately connected to digestive health.^42^

This presentation of the stomach as a barometer of psychological strain rested upon a complex set of manoeuvres. First, a series of practical interventions allowed the form and content of the stomach to be measured in different situations. From the end of the nineteenth century, a whole host of technologies had emerged—the stomach bucket and gastrograph, Ryle’s ‘gastro-investigative tube’, the X-ray and the fractional test meal—that together combined to reveal the occult workings of the stomach and render them quantifiable.^43^ These new technologies did not demonstrate a straightforward correspondence between the actions of the stomach and the process of digestion. Experiments on patients with open fistulas and upon laboratory animals such as Pavlov’s ‘pouch dogs’ (creatures with artificial pockets created along their digestive tracts for the collection of internal secretions) indicated that the production of pepsin and gastric acid did not simply correlate with episodes of feeding and digestion.^44^ In most cases, the promise of food and its associated visual and olfactory cues would trigger a sequence in which peristalsis was initiated and digestive enzymes produced; however, in anxious or frightened animals, this process was disrupted. The Harvard physiologist, Walter Cannon, an early pioneer of psychosomatic approaches, noted that the production of gastric juice did not occur when frightened laboratory cats were offered food, but quickly returned on stroking.^45^ The disparity between the stomach’s actions and the animal’s situation was explained by introducing another order of time.^46^ The stomach was not simply responding to the external cues of offered food or threatened starvation; rather, it was rehearsing primitive responses to perceived threats.^47^ As Walter Alvarez, author of the classic work, *Nervous Indigestion* (1930) noted, ‘these nervous inhibitions, of little use to us today, are survivals from our cave-dwelling forebears whose lives at any moment might depend on the strength that could be withdrawn from the inner organs and concentrated in muscles needed for fighting or running away’.^48^ This temporal disjuncture was heightened in those involved in transport work. In 1930, Harold Dodds, a surgeon at the Royal Marsden Hospital, had written to the *BMJ* noting high rates of peptic ulceration in busworkers.^49^ By 1935, Walter Langdon-Brown, the Regius Professor of Medicine at Cambridge, could claim that it is ‘common knowledge that chauffeurs and omnibus drivers are especially liable to the condition [gastritis]’ since ‘the state of chronic tension induced by the traffic of London streets must also be a powerful agent’. This tension emerged because of the temporal disjuncture. It was a result of old time persisting in new bodies. As Langdon-Brown explained: ‘The autonomic nervous system … mobilises man for action, but under modern conditions the primitive reaction is repressed. Hence the steam of energy is spent internally’.^50^

The stomach in combination with medical technologies thus enfolded different orders of time. It brought together the lived time of modernity and the ancestral time of evolution and in doing so, it opened up the possibility of a new form of critique. The legitimacy and acceptability of modern working practices could now be judged against the composure of the stomach. The artificial time of the worker’s day was benchmarked against the natural rhythm of the digestive system. The validity of this political operation was underlined by a growing epidemiological literature that demonstrated the uneven distribution of gastric disturbances across age, class and occupation.^51^ Epidemiological investigations correlated gastric disruption and social disruption within both the space of individual lives and across historical time. By the end of the 1930s, it was widely believed that the British population was undergoing a psychosomatic transition. Older and more dramatic forms of psychological distress (such as hysteria) were being replaced by more subtle neuroses that manifested themselves in somatic form. Among these neuroses, the gastric disturbances loomed large.^52^

How are historians to understand this transformation? At one level, it is of course tempting to overlook physiological questions and instead read these concerns in cultural terms. Certainly, it is possible to argue that rising concern over the increase in gastric disorder can be seen as a kind of self-fulfilling bodily prophecy or, to use Ian Hacking’s phrase, a ‘looping effect’.^53^ It was an inter-war commonplace that the English had become a nation of ‘stool collectors’ and gastric obsessives.^54^ Massive investment in adverts for stomach powders and laxatives in the 1920s and 1930s fostered this culture of concern.^55^ This could have encouraged a ‘new somatic mode of attention’ in which more notice was paid to the movements of the stomach, its fleeting pains, the timing of defecation and the consistency of the excreta: a process which in turn would lead to the product of new bodily feelings.^56^ However, while such readings are tempting, we should recognise the political costs of this cultural turn.^57^ Reducing busman’s stomach to a cultural epiphenomenon is a political intervention. Writing off such conditions as ‘all in the mind’ closes off specific pathways of remedial action and undermines the patients claim. Diagnosis is a political action.^58^ Recognising the complex origins of busman’s stomach at the intersection of labour, belief, medicine and technological practice shows how it cannot easily be resolved into our familiar organising categories of nature and language. And indeed, it was busman’s stomach disputed existence on the uneasy boundary between biology and culture that gave shape to the course and outcome of the Coronation Bus Strike. It was, to return to Saler and Bennett’s arguments, a kind of magical object. Conjured out of insurance claims and new medical instruments, it brought together different orders of time to make possible a new kind of political action.

## The politics of Busman’s stomach

Busman’s stomach first emerged at the centre of a number of competing claims around injury, illness and industrial compensation. In July 1931, J. R. Clynes, the Labour Home Secretary, requested that the Committee of Inquiry on Workmen’s Compensation extend its investigation to cover the possible scheduling of carbon monoxide poisoning.^59^ This was not a straightforward process. Carbon monoxide is an unstable entity and earlier governmental investigations had demonstrated the difficulty of assessing atmospheric carbon monoxide and carbon monoxide poisoning at low levels.^60^ Moreover, the symptoms associated with poisoning were inconsistent. Called before the Committee, Ambrose Woodall, the Physician at Manor House (the Trade Union Congress Hospital), testified that busmen commonly suffered from a number of conditions that might be brought about by ‘constant inhalation of small quantities of CO—debility, neurasthenia, cardio-vascular and gastric symptoms’.^61^ Busmen, in particular, he argued, were more likely to present with gastritis—with the condition occurring in 34 per cent of patients compared to an average of 15 per cent in all other groups. The sheer multiplicity of symptoms and the lack of any objective measures meant that no illnesses could be attributed with any certainty to low-level carbon monoxide poisoning.^62^

The failure to connect carbon monoxide poisoning to digestive disturbances left gastritis as an orphaned symptom. Although the association between buswork and gastric illness seemed strong, the changes made visible through barium meals and insurance returns were robbed of their meaning. In the absence of reliable diagnostic tests that could demonstrate the origin of digestive disturbances in carbon monoxide poisoning, the disorder began to be seen in psychosomatic terms—as an index of the strain of modern working conditions. Following the publication of the Second Report on Workmen’s Compensation, Bevin began to lobby for a new investigation into rates of gastritis in busworkers, encouraging Sir David Munro, the Secretary of the Industrial Health Research Board (IHRB), to reopen his discussions with Woodall.^63^ Although he recognised that the attempt to schedule gastritis as a symptom of carbon monoxide poisoning had failed, Bevin argued that gastritis on its own could be seen as evidence of pathological strain and the irregularity of the busman’s life.^64^ Gastritis moved from being a symptom of gas poisoning to become an illness in its own right.

Although members of the IHRB were willing to concede that buswork might be a source of nervous strain, they were sceptical about the possibility that such strain could ever be quantified.^65^ This position was widely held. Major Greenwood, perhaps the main promoter of a statistical approach to epidemiological investigation in inter-war Britain, complained that although it was obvious that city life had a pathological effect, the actual speed and stress of modern existence remained incalculable.^66^ As Greenwood confessed: ‘I do not myself know of any way in which the “strain” or “pace” of life in towns, or alternatively, of modern life … can be measured, one difficulty, being no doubt, that nobody has yet defined these things in an unambiguous way’.^67^

Greenwood’s solution would be supplied by the busmen’s bodies in combination with the theoretical tools of two of his protégées at the London School of Tropical Hygiene and Medicine, the psychologist, Millais Culpin and the statistician, Austin Bradford Hill.^68^ The IHRB consulted with each of them during their initial discussions into the possibility of an investigation. Both had long-standing relationships with the Board and both brought a particular set of methods and perspectives that together would make busman’s stomach visible. Culpin had carried out a number of investigations for the IHRB into the relationship between work strain and physical reactions.^69^ He claimed that seemingly neurotic conditions were better understood as forms of minor psychoses: psychological reactions held in place and sustained through a mixture of fear, stress, ambition and pecuniary interest.^70^ In Culpin’s writings, the concept of the dynamic unconscious worked to draw together a host of disparate complaints and turn them into symptoms of a single psychopathological condition.

Hill shared Greenwood’s commitment to Karl Pearson’s expansive vision of statistics and the conviction that old ideas of mechanical causality could be replaced with a broader conception of correlation ‘between two occurrences embracing all relationships from absolute independence to complete dependence’.^71^ Pearson had maintained a nominalist approach to these relationships, arguing that experimental phenomena should be seen as working abstractions that were lent credibility through their mathematical demonstration.^72^ Greenwood and Hill shared this position, but this cautionary nominalism was lost in the political discussions that followed Hill’s investigations. Indeed, statistics and psychology would become the instruments that materialised busman’s stomach and granted it political force.

In March 1934, the Medical Research Council (MRC) Statistical Committee agreed that Hill should undertake a preliminary investigation into the incidence of gastritis in busmen, although it noted that such an investigation would be fraught with difficulty. Hill shared their scepticism, noting that the sickness records kept by the approved societies administering national health insurance in London transport workers were patchy and that it was difficult to imagine what kind of control group could be found.^73^ Like Greenwood, he insisted that any attempt to develop a statistical measure of the strain of modern existence was doomed to failure, yet the net effect of his careful investigations was to turn the busman’s body into an instrument that would display through its gastric pathologies the cumulative force of city life.

This was never his intention. Hill had always maintained an agnostic position on the busmen’s claims.^74^ Indeed, he presented his investigation as a form of ‘flapdoodle’—a test of the beliefs of union leaders rather than the prevalence of a disease.^75^ He argued that the rates of digestive sickness recorded in the insurance records were the ‘end result of many causes, the separate influences of which are not measurable’.^76^ These causes included the particular demands of the work, the age of the workforce and rates of sickness benefit payable to employees as well as the folk beliefs about sickness held among the workers.^77^ The sheer mass of confounding variables made the choice of control group difficult. Hill identified London Transport indoor staff, ASLEF and NUR members before deciding that the only reliable comparator for bus drivers would be tram drivers, since they shared the same employers, age profile and insurance scheme.^78^ They presented a kind of natural experiment with the division of labour helping force workers into groups that could be used for comparative analysis. His choice was backed by TGWU representatives who claimed that it was common knowledge that tram drivers were somehow protected from the problems that confronted the busmen.^79^ However, the busmen’s representatives complained that while the tramworkers may have provided a good point of comparison in measuring the effects of carbon monoxide—since the trams were electric—they still faced the same problems of nervous strain that confronted the busmen.

Hill’s preliminary figures, leaked from meetings between the MRC and representatives of the TGWU, suggested that there was a slightly higher incidence of gastric disorders in middle-aged bus drivers. In drivers aged between 40 and 49, 16.3 per cent of all days lost through sickness were attributed to digestive disturbances as against 13.5 per cent among tramwaymen in the same age group.^80^ Although the proportional difference was small and despite the fact that Hill himself had scrupulously avoided speculation over possible aetiological mechanisms, the sheer fact that he had begun to abstract cases of gastritis and collapse them into a single category for comparison turned what had been a symptom in many different illnesses (ranging from peptic ulcer to influenza) into a single illness associated with a single occupation.

The TGWU and the RFM worked hard to connect the disorder, abstracted in the MRC’s statistical investigations, to the conditions of modern labour. In December 1936, as Hill was completing his study, John Langdon Davis, the science correspondent of the *News Chronicle*, published a scoop on the ‘strange illness of London bus conductors’ urging the public to have more sympathy with bad tempered ticket collectors since the strain of irregular meals and toilet breaks created a situation in which ‘no human nerves can be expected to control either the gastric juices or the temper’.^81^ Within the space of a few months of the leak, Hill’s epidemiological analysis would find itself reworked into syndicalist pamphlets, parliamentary questions and folk protest songs. In April 1937, the RFM published *London Busmen demand the right to live a little longer.* They complained that that the tremendous strain imposed upon the health and nervous systems of the busmen was turning A1 men into C3 scraps.^82^ Drawing upon Hill’s figures and the records held by the London General Omnibus Company Employees Friendly Society, they argued that only 10 per cent of busmen reached retirement, with one-third being discharged due to ill health at an average age of 46 and 20 per cent dying in service.^83^ The pamphlet sold over 250,000 copies.^84^ Public awareness of the epidemiological costs of buswork was furthered through protest songs. Radically marrying morbidity statistics and folk music, the busmen composed a lament based upon Hill’s figures to the tune of Clementine:

London busmen stick together

This is your right to see it through

For though buswork may be thrilling

We can prove it’s killing too

Only four men in a hundred

Reach the age of sixty-five

Whats the use of having a pension

Unless you’re still alive?^85^

The busmen’s argument rested upon the correlation of the health decline revealed in the Friendly Society statistics with the broader transformation of the worker’s environment. It was an argument that exploited, and in some ways subverted, the paper culture of the LPTB administration. The steady decline of the busman’s health was objectively demonstrated through reference to the company’s personnel selection tests and the medical certificates demanded under the Metropolitan Police licensing scheme for Drivers and Conductors.^86^ Likewise the idea of a transformed environment, an idea that had been a familiar yet somewhat nebulous bugbear in patrician critiques of speed, could be traced in the annual reports produced by the LPTB and the Ministry of Transport, and in the vast armamentarium of technologies and schedules that the Board produced to regulate driver performance.^87^ It could be shown that the 1932 agreement had brought about an increase in average driving speed (from 9.07 mph in 1928 to 10.42 in 1936–1937) while at the same time, traffic conditions became more intense: new model buses carried almost twice as many passengers and the density of recorded central London traffic more than doubled between 1919 and 1935.^88^ The 1934 Road Traffic Act exacerbated these challenges through the introduction of traffic lights and pedestrian crossings. The stress of these changes, incarnated in the busman’s body and the LPTB’s papers, formed the basis of the busmen’s claim for a seven-hour day.

Although the flesh and paper bases of the busman’s claim for a seven-hour day appeared strong, it was difficult to implement in practice, given the difficulties of coordinating shift times and schedules. From the outset, Bevin persuaded members of the RFM to moderate their initial claim, arguing that actual duties would work out at only six hours under their scheme. ^89^ Despite this concession, Ashfield and Pick refused to entertain the idea of any serious easement of the workers’ conditions.^90^ On 31 March, after four-and-a-half months of fruitless negotiation, the TGWU gave notice that it intended to suspend the 1932 agreement. The strike began on 1 May and met with 100 per cent support. Three days later, the Ministry of Labour ordered the formation of an Industrial Court of Inquiry: a form of tribunal that Bevin had successfully exploited during the 1920 Docks dispute.^91^

Originally established during the First World War as a public forum through which the state could arbitrate trade disputes, in the 1937 Coronation dispute, the Industrial Court of Inquiry came to resemble a scientific commission. Medical experts from the IHRB and Manor House were called to give evidence on the existence of busman’s stomach and its possible aetiology. Despite the central role granted to scientific expertise, Greenwood and Hill remained sceptical about the meeting’s potential, noting in private that ‘A Court or Committee Enquiry, which commands the public confidence, as the phrase runs, is usually totally incompetent’. The only hope lay in obtaining ‘a judge of the High Court as chairman, restrict membership to a physiologist, a physician, a statistician and a representative of the Government Actuary’^92^

In the absence of this kind of expert tribunal, the Industrial Court of Inquiry was transformed into a contest between competing lay understandings of human nature and temporality. It can be seen, to borrow Bruno Latour’s phrase, as a ‘parliament of things’: a forum in which the existence of certain objects was contested but which in turn found its actions and decisions shaped by those selfsame objects.^93^ The Court of Inquiry was coordinated around the contest between two objects: the 1933 LPTB stockholder agreement and busman’s stomach.^94^ In the negotiations that had preceded the strike, Pick and Ashfield had claimed that their discretion over wages and timetabling was restricted by their need to pay a set annual dividend on B stock shares to the former owners of Underground Electric Railways and the London and Suburban Companies.^95^ Failure to make these payments would lead to the removal of the directors and the appointment of receivers. Bevin sought to demonstrate that this constraint was illusory, arguing that the stockholder agreement could be renegotiated and that the rates of return had been artificially inflated by the concessions and profits won by the companies in the Speed Up agreement of 1932.^96^

Speed would serve a twofold role in Bevin’s rhetoric. It undermined the objectivity of the stockholder agreement and underlined the reality of busman’s stomach. At the same time, as it revealed the artificially inflated basis of the stockholder agreement, it demonstrated the exploitative origins of the busman’s gastric conditions. Bevin achieved this by marrying the statistical and experimental evidence produced in the MRC investigations with new understandings of evolutionary temporality. He argued that the bus industry threw up unique problems in governance because even though working hours may be limited, those limited hours were experienced at increasing intensity.^97^ This intensity, sustained through the discipline of the rule book, traffic circulars and rotas that appeared like ‘Japanese writing shorthand’, combined with heavier and more complex traffic conditions to produce a new kind of individual.^98^ As Bevin noted:

The physical reactions of the busmen are such that they have presented to us, the more rapid evolution [*sic*] a complicated reaction that has made the men themselves different from the men found in other industries, different in the sense that it has produced a pathology all of its own.^99^

This evolutionary pressure affected both passengers and staff. As Bevin remarked during his cross-examination of Ashfield; ‘I sometimes wonder, Lord Ashfield, what kind of animal you are going to produce in the end with all the strap-hanging in buses and tubes, what with balancing tricks and all the crushing. I wonder what the future man and woman will be like’^100^ The regime of buswork imposed by the LPTB schedules was presented in Bevin’s rhetoric as a kind of evolutionary experiment. Busman’s stomach demonstrated that that experiment had failed.

In Bevin’s evolutionary arguments, busman’s stomach served as a pathological metric, demonstrating the tension between natural and industrial time. Yet, the object was inherently unstable. Sustained, as it was, by a framework of interlocking theories and technologies, the phenomenon could always be unpicked in an adversarial setting by undermining the accuracy of specific techniques or by positing alternative causes for the phenomenon. In his opening statements, Bevin admitted that he remained agnostic as to the precise mechanism that lay behind the disorder: ‘I have no prejudged opinion whether it is intensification, or the type of food, or whether it is eating food too quickly, or whether it is the irregularity of habits, I am not a doctor, but one has to investigate a good many of these things in conjunction with experts and it may be a simple remedy for it may be found if you can get a cause’.^101^ At the same time, he did not doubt the reality of the condition, using the worker’s everyday experience of gastric sickness to trump any evidence produced in the board’s statistical investigations. ‘I do not think I am exaggerating when I say that my members who are employed on buses and other vehicles in London are extraordinarily good customers of patent medicine merchants. They get this flatulence and gastric troubles and they try to ease it and I do not think they are getting good results for their money because you are not touching the seat of the problem’.^102^

Despite his contempt for the Board’s statistical approach, Bevin drew upon actuarial work comparing rates of sickness among members of the Licensed Vehicles Workers Sick Benefit Club with the averages produced by the more general membership of the Manchester Unity.^103^ At the same time, medical witnesses including Ambrose Woodall (of Manor House) and Hyacinth Morgan, Medical Adviser to the TUC, testified to the reality of the condition. Woodall argued for the objective basis of the disorder on clinical grounds, explaining that at Manor House ‘we submit the patients to fractional test meals, to x ray examinations, and to examination of the stool to make certain, as far as possible, that the condition is either a gastric or duodenal ulcer’.^104^ Morgan pursued the statistical argument, noting his own studies of admissions which he linked through guarded references to Hill’s investigation. In his cross-examination of their arguments, Pick sought to demonstrate that the illness was an artefact: a fabrication created through the division of clinical labour in medical inspections and sustained as collective fantasy among the men.^105^ Thus, he acknowledged that ‘this widespread gastric trouble matter had been much discussed among the men for some number of years’ but argued that ‘talking about a particular class of a disease rather encourages the symptoms of that disease’.^106^

Despite Pick’s efforts, the Court did not dismiss busman’s stomach. Although they rejected the old arguments around CO poisoning and the effects of vibration, the Inquiry’s chairman, John Forster, acknowledged, the apparent increase in nervous tension. He echoed Bevin’s evolutionary concerns, stating that the Board was ‘engaged upon making a biological and mechanistic rhythm harmonise and that must necessarily set up a strain in the human body and mind’.^107^ However, it was difficult to assess the effects of such strain. He noted the IHRB inquiry into busman’s stomach but suggested that this needed to be supplemented by additional work on the illness’s causation.^108^ A new committee was convened under Forster’s chairmanship, comprising representatives of the MRC (including Bradford Hill), the LPTB and the TGWU. The moral struggle that lay behind the busmen’s claim was transformed into a technical debate over the methods of statistical inquiry.^109^

In the wake of this inconclusive inquiry, the union executive’s attempts to reach a settlement with the LPTB were repeatedly rejected by the busmen’s delegate conferences.^110^ Faced by this recalcitrance, Bevin and the General Executive Committee took direct control of the dispute. On 25 May, they decided to revoke the powers of the Central Bus Council, ordering the men back to work the next day.^111^ On 7 June, members of the Bus Section were informed that the powers of their Central Committee, District Committees and Delegate Conferences had been suspended, pending an investigation into the role of unofficial movements.^112^ Five days later, Papworth, Payne and Jones were formally expelled from the union and their colleagues in the Rank and File organising committee were debarred from holding office for two years.^113^ On 15 June, Bevin sent out a circular to all members of the London Bus Section detailing the modest gains of the new agreement.^114^ Eight months later, encouraged by the maverick politician, William J. Brown, Bill Payne along with 10 of the 150 branch officers, joined the breakaway National Passenger Workers Union.^115^

Although the Coronation Strike ended in disarray and recrimination, it still served to change the grounds of industrial debate. Busman’s stomach and the model of social justice that it sustained now entered the popular imagination. Patent medicines and political programmes all referenced the condition.^116^ Left-wing papers, such as *Reynolds News,* carried human interest stories describing the plight of busmen’s wives, seduced by the glamour of uniform only to find themselves nursing nervous wrecks.^117^ The *Daily Sketch* and *The Times* which had consistently editorialised against the busworkers as a disruptive and radical menace conceded that the ‘human considerations must take precedence of financial cost if it is to be found that health is injured by nervous strain and physical wear and tear of driving to timetable through London’s crowded thoroughfares.’^118^ The *Spectator* concurred, acknowledging the reasonableness of the busmen’s case and demanding the repeal of the London Passenger Transport Act if the workers’ health continued to be sacrificed to shareholder demand.^119^ During the dispute, the London Passenger Board was inundated with letters from members of the public protesting the injury to the busman’s health.^120^

In February 1938, the socialist Unity Theatre company returned to the Coronation Strike for its first ‘Living Newspaper’ production. Directed by John Allan and scripted by the young communist Montagu Slater, the play mixed techniques drawn from American documentary theatre, Mass Observation, Soviet realism and German expressionist ballet in its attempt to capture the pathological effects of modernity on individual health.^121^ Statistics of increased industrial production were projected onto a stage screen, while spotlights picked out the figures of sick and broken busmen. The loudspeaker’s narration of the successes of amalgamation and occupational acceleration was interrupted by the plaintive monologues of the drivers describing their nervous collapse. Modern driving, they protested, ‘Was like trying to sleep in a Wurlitzer factory’. The play, as Robin Jardine informed readers of the *New Left Review,* was a clear demonstration of the high cost of ‘savings squeezed out of over-wrought nerves, out of ulcerated stomachs, out of men made old before their time so that only one man in 11 reaches the age of 65’.^122^ The play reflected the strained temporality of modern buswork by abandoning conventional staging to compress 18 scenes, ranging from Nunhead to Westminster, into an hour-long production. Likewise, it reflected the union’s commitment to the materiality of the busmen’s complaints. As the writers explained, ‘Every emphasis was laid throughout on the reality of the theme’ with fragments from Court of Inquiry and widely reported deathbed speeches taking on a new life as public entertainment.^123^

The shifting meaning of gastritis—from a sign of gas poisoning to symptom of exploitation and inequality—was confirmed by the communist biologist, JBS Haldane.^124^ Writing in the *Daily Worker* in 1939, Haldane noted that ‘The commonest cause of gastritis—that is to say an inflamed and irritable stomach—is worry and anxiety. It is particularly common among busmen and travelling salesmen. I had it for fifteen years until I read Lenin and other writers, who showed me what was wrong with our society and how to cure it. Since then I have needed no magnesia’. Although he warned his readers that serious stomach pains should always be investigated by a qualified doctor, the typical GP would probably just recommend laxatives or antacids, whereas, he added ‘the *Daily Worker* may effect a permanent cure’.^125^

At the start of this paper, I noted how historians have described the twentieth century as a period of re-enchantment. In Haldane’s attempt to restore gastric equilibrium through a programme of political education, I think we can see a kind of magic at work. Alongside the sorcery that blended together political belief and physiological transformation, we can recognise in this history the ways in which new objects are conjured into the world. In the mundane administration of sickness club claims and annuity returns, we see a process which inverts Marx and Engels’s dictum that ‘all that’s solid melts to air’. Here, fleeting experiences and accidents were being magicked into concrete objects. And these objects had the power the change the world. As the Forster Inquiry demonstrated, the magic of these objects only became effective once the complex labour that underwrote their construction—the harnessing of clinical technique, psychological argument, statistical methods and models of temporality—was obscured.

In the end, busman’s stomach would provide an unstable foundation for political argument. When Forster’s new committee reached its conclusion in 1939, gastritis was no longer seen as an index of modernity, but rather as a product of a particular kind of personality.^126^ It was a conclusion that Hill’s colleague, Millais Culpin had been advancing since the beginning of the inquiry.^127^ A second MRC investigation, conducted by L G. Norman into the role of occupational factors in the aetiology of peptic ulceration 1951, revealed little difference in ulceration rates between professions, but instead suggested that buswork might instead attract a particular type of stress-prone personality—’the driving character’.^128^ Busman’s stomach was transformed from an illness of labour conditions to become instead a psychological characteristic. In the post-war era, the busman’s claim for social justice would find itself transmuted into a case for individual therapy.

## Notes on contributor

***Rhodri Hayward*** is a reader in History at Queen Mary, University of London and a co-founder of the Queen Mary Centre for the History of the Emotions. He has published on the history of dreams, Pentecostalism, demonology, cybernetics, and the relations between psychiatry and primary care. His current research examines the rise and political implications of psychiatric epidemiology in modern Britain. His book Resisting History: Popular Religion and the Invention of the Unconscious was published by Manchester University Press in 2007 and The Transformation of the Psyche in British Primary Care by Bloomsbury in 2014.

## Funding

This work was supported by the Wellcome Trust [WT grant number 082971].

## Notes

1. ‘London Bus Strike: 27,000 Workers Involved’, (*The Times*, May 1, 1937), 16a. For overviews of the strike, see: Fuller, *Radical Aristocrats*, 148–156; Glatter, ‘London Busmen’; Clegg, *Labour Relations*, 120–156; Barker and Robbins, *History of London Transport,* vol. II, 325–328; Smith, ‘Return of the Rank and File’.

2. Bullock, *Life of Ernest Bevin* vol. 1, 521–523, 606–614; Fishman, *British Communist Party,* 118–125; Weller, *Ernest Bevin,* 84–86.

3. Bill Jones *Busworker’s Monthly* (May 1983), quoted in Fuller, *Radical Aristocrats*, 156; Fishman, *The British Communist Party and the Trade Unions, 1933*–*1945*, 118–125. See also the protest letters sent to Bevin held at Warwick University Modern Record Centre (MRC) MSS 148/A26 Leo Little (2 June 1937); H. R. Selmes (2 and 4 June 1937); A. J. Pope (22 June 1937); E. Stafford (22 June 1937); W. G. F. Searl (4 June 1937).

4. Glatter, ‘London Busmen’, 10.

5. Fuller, *Radical Aristocrats,* 154–155. The same claim is made in Barman, *Frank Pick,* 158–159; German and Rees, *People’s History of London,* 196–197; Hibbs, *British Bus Services,* 152–153; Wrigley, ‘Trade Unions’, 86–87.

6. For the critique of ‘rank and filist paradigm in historiography, see: Zeitlin, ‘Rank and Filism’ and responses by Cronin, ‘Rank and File’, Price, ‘What’s in a Name?’.

7. Thompson, ‘Moral Economy’. I have discussed this shift in Hayward, ‘Pursuit of Serenity’.

8. Rabinbach, *Human Motor,* 220–224; Cross, *Quest for Time,* 111–120.

9. For overviews: Vernon, *Distant Strangers;* Berman, *All that’s Solid;* Giddens, *Modernity and Self Identity;* Eisenstadt, ‘Multiple Modernities’.

10. Polanyi, *Great Transformation*; Weber, *Protestantism.*

11. Weber, ‘Science as a Vocation’, 139. Bennett, *Enchantment*, 56–60.

12. Marx, ‘Speech at the Anniversary of the People’s Paper’.

13. Saler, ‘Modernity and Enchantment’; Owen, ‘Occultism’, Bennett, *Enchantment*.

14. Latour, ‘Sampling the Soil’, 58, 71–72; Pickering, ‘Alchemy’.

15. My reading here is pretty heavily indebted to Bennett, *Enchantment*, chaps. 5, 6 and 8.

16. Renshaw, *Busmen’s Rank and File.*

17. Renshaw, *Busmen’s Rank and File,* 11; Glatter, 6; Fuller, 113.

18. Renshaw, *Busmen’s Rank and File Movement,* 6; Corfield, ‘Union No. 29’, 43.

19. Hutt, *Post War History,* 235–236; Fishman, *British Communist Party,* 51–54.

20. Renshaw, *Victorious Strike,* 8.

21. Fuller, *Radical Aristocrats,* Chaps. 10–11. McIlroy, ‘Revival and Decline of the Rank and File’ attributes much more influence to the CPGB, see: 352–353.

22. Clegg, *Labour Relations,* 14–30; Fuller, *Radical Aristocrats,* 67–71.

23. Corfield, ‘Union No. 29’.

24. Renshaw, *Busmen,s Rank and File, 6;* Corfield, Union no. 29’, 43.

25. ‘Five Hundred Busmen on Strike against Speed Schedules’, (*Daily Worker*, January 19, 1933), 3.

26. Renshaw, *Victorious Strike,* 5.

27. Marriot, ‘Sensation of the Abyss’, 85.

28. Morrison, *Socialisation and Transport.*

29. Barker and Robbins, *London Transport II,* chap. xv.

30. Hibbs, *British Bus Services,* 195–196; Hurcomb, ‘Co-ordination of Transport’; BLPES Omnibus Strike vol. 8: John Cliff, The London Passenger Transport Board MS.

31. Barman, *Frank Pick,* 154–159.

32. For an overview of Pick’s thoughts on speed, evolution and entropy, see: Barman, *Frank Pick,* 161–164; Saler, *Avant Garde,* 118–121. Saler has argued that Pick’s vision became increasingly pessimistic following his move to the LPTB and his private recognition of his own limitations. However, his public commitment to speed continued unabated.

33. London Transport Museum, FP A Studd Lecture, 21.

34. Eddington, *New Pathways in Science,* 55–66; Barman, *Frank Pick,* 166.

35. LTM Frank Pick Collection: PA33: ‘Some Reflections on the Administration of a Public Utility Undertaking’.

36. [LPTB], *Proceedings Second Annual Conference,* 24–25.

37. The last popular reference I can find is in 1969 (*On the Buses* series 1 episode 4) see Walker, *On the Buses,* 55.

38. Overview given below. Bevin in evidence to the Industrial Court of inquiry 3 May 1937, 10 dates interest in the question back to 1928. BLPES: Omnibus Strike vol. 1, AB400755.

39. Llewelyn Smith, *New Survey of London* VIII, 84. On Smith’s survey, see: Alexander, ‘A New Civilisation?’

40. Johnston, *Morbid Sensibility,* 102–103; Stephen Shapin, ‘Trusting George Cheyne’; Williams, ‘Pinel’.

41. For a good overview, see: Miller, *History of the Stomach,* 19–31; Jonnson, ‘Physiology of Hypochondria’, 15–30.

42. See Miller, Jones, ‘Gut War’. Walton, ‘Peristaltic Subjects’, Hayward, ‘Halliday’.

43. See Arthur Hurst, ‘Introduction’ to Bengt Ihre, *Human Gastric Secretion,* (London: OUP, 1938). For a useful list of new technologies, see Morton, *Diseases of the Stomach,* chap. 11; Knud Faber, *Gastritis,* 22–32, 69–73.

44. Davies, ‘Nervous Dyspepsia’, 152–153; Davies, ‘Some observations on peptic ulcer’. For overviews of the new science and the ways that gastric disruption was read as a sign of emotion, see: Alvarez, ‘Digestive tract’, 1231–1237; Flanders Dunbar, *Emotions and Bodily Changes,* 293–305; Wittkower, ’Influence of emotion’, 561–606.

45. Cannon, ‘Movements of the Stomach’; ‘Influence of Emotional States’; 5–6. *Mechanical Factors of Digestion,* 217; *Bodily Changes in Pain,* 12–16, 253–254; *Digestion and Health,* 125–126 For overviews of Cannon’s work see: Dunbar, *Emotions and Bodily Changes,* 272, 293–294; Young, ‘Walter Cannon’, 234–256; Jackson, *Age of Stress,* 70–96.

46. I am condensing a more complex literature on the role of technology in transforming inner experience, see: Dunbar, *Emotions and Bodily Changes*, 107–108; and for analysis Dror, *Modernity and Scientific Study,* 178, 181–185. For a suggestive account of how electrophysiological techniques served to disrupt conventional ideas of temporal sequence, see: Schmidgen, *Helmholtz Curves,* 14–16, 85–104, 173–175.

47. On the connection of physiological time and evolutionary time, see: Young, *Harmony of Illusions,* 21–27; Hayward, ‘Darwin’s Changing Expression’.

48. Alvarez, *Nervous Digestion,* 26.

49. Dodds, ‘Perforating ulcer’, 265.

50. Langdon-Brown, ‘Visceral Symptoms’, 615, 617.

51. For early accounts of the occupational basis of gastric disorder, see: McCarrison, ‘Address on faulty food’; Hawkins, ‘The Reality of Enterospasm’; Davies and Wilson, ‘Observations on Peptic Ulcer’; Barford, ‘Statistical inquiry. For a discussion of the post war literature, see: Jones, ‘Stomach for Peace’.

52. For discussions of the increase in gastric disturbance see: Halliday, ‘Incidence of psychosomatic affections’. For commentaries, see: Edgar Jones, ‘Gut War’; Hayward, ‘Enduring Emotions’, 827–838.

53. Hacking, *‘*Looping Effects’; Hacking, ‘World-making by Kind-Making’.

54. Hutchison, ‘Hypochondriasis’, 365; Guirdham, ‘Constipation’.

55. Indigestion cures only appear as a separate category in the *Statistical Review of Press Advertising* from volume 5 no. 1 (October 1936). But by the first quarter of 1937, £94, 688 was being spent advertising indigestion cures (£333,732 between Oct 1936 and Oct 1937). For a good guide to the way that cultural agendas shape kinaesthetic experience, see: Leder, *Absent Body.*

56. Csordas, ‘Somatic Modes of Attention’; Hinton and Simon, ‘Cutlural Neuroscience of Anxiety Disorders’.

57. For a good non-culturalist assessment of the relationship between the conditions of driving and new emotional and physical states, see: Michael, ‘Co(a)gents and control’.

58. White, *Sustaining Affirmation,* chap. 1.

59. Departmental Committee on Compensation for Industrial Diseases, *Second Report,* 10 (evidence of Dale Logan).

60. 
*Final Report of the Departmental Committee on Ethyl Petrol,* 78–87; Haldane, ‘Carbon Monoxide Poisoning’.

61. 
*Second Report on Workmen’s Compensation,* 1933, 13–14.

62. 
*Second Report,* 14–17; TNA FD1/4082 Lansborough Thomson to David Munro (15/11/1934).

63. TNA FD1/4082. David Munro to Ambrose Woodall 20 November 1933.

64. This rhetoric equating gastritis with the strain of speed was echoed in the writings of the Rank and File movement, see: Warwick MRC MS 62/4/3/14 [Nipper], ‘Some points about bus work’, *Busman’s Punch* 18 ns (May 1934); MS 62/4/3/14 [Editorial], ‘The Seven Hour Day’, *Busman’s Punch* 19 ns (May 1934); ‘Let’s have a moan’; MS 62/4/23 ‘Speed and Health’ *Busman’s Punch* 35 ns (June 1935).

65. David Munro to Ernest Bevin 7 December 1933, TNA FD1/4082.

66. On Greenwood, see: Matthews, *Quantification;* Hardy and Magnello ‘Statistical methods’; Magnello, ‘Mathematical statistics’, 95–123.

67. Greenwood, *Epidemics and Crowd Diseases,* 135.

68. Higgs, ‘Medical statistics’; Farewell, et al., ‘Memorandum’; Culpin, ‘Autobiography’.

69. Culpin, ’Nervous disease’; ’Need for psychopathology’; ‘The nervous temperament’; *The Nervous Temperament.*

70. Culpin, ‘The conception of “nervous disorder”’*,* 1383–1387; ‘Some cases of “traumatic neurasthenia”’, 233–237; Culpin, ‘The Problem of the Neurasthenic Pensioner’, 316–328; Figlio, ‘How Does Illness Mediate Social Relations?’, 174–224.

71. Pearson, *Grammar of Science,* 157.

72. On Pearson’s nominalism see: Derosières, *Politics,* 105–112.

73. MRC Statistical Committee Minute 324 (30/4/1934); IHRB Minute 267 (26/111934). TNA FD3/79; Hill, *Investigation into Sickness Experience,* 8–9. TNA FD1/4082 David Monro to Frank Stillwell, (26 April 1934); Austin Bradford Hill to Frank Pick,.

74. For an early statement of Hill’s scepticism, see: [A. B. H.], ‘Review of *New Survey’.*

75. TNA FD1/4082 Austin Bradford Hill to David Munro (1/11/1934).

76. TNA FD3/79 Austin Bradford Hill, *An Investigation into the Sickness Experience*, 3.

77. 
*Sickness in Transport Workers,* 2; FD1/4083 Gastritis in Busmen II: M. Harvey to D. Munro (1/7/1935).

78. TNA FD1/4083 IHRB Committee on Sickness amongst Omnibus Workers Minutes of First Meeting (21 May 1935); Hill, ‘Sickness among Transport Workers: Memorandum No. 2’; IHRB Committee on Sickness amongst Omnibus Workers Minutes of First Meeting (5 December1935): Hill, *Principles.* chap. xv.

79. Hill, *Experience of Sickness*, 4–5; TNA FD1/4083 IHRB Committee on Sickness amongst Omnibus Workers Minutes of Second Meeting (11 October 1935) [interventions by Harold Clay and Frank Stillwell].

80. TNA FD1/4084 Gastritis in Busmen III A. B. Hill, Supplement to Memorandum no. 5. See also: *Forster Inquiry*, 4 para 7.

81. [John Langdon Davies]. ‘Strange Illness of Bus Conductors’ *News Chronicle* (2 December 1936), 2.

82. 
*London Busmen demand,* 9.

83. For detailed criticisms of these claims see: Forster Inquiry (1939), 12–13; paras 13, 14.

84. Corfield, ‘Union No. 29’, 46.

85. Peter Glatter, ‘London Busmen’, 6.

86. Dunlop, ‘Medical Standards’; see also Harman, ‘Vision and the Motorist’; *Public Service Vehicles.*

87. The most notable exponent of this patrician critique was Stanley Baldwin, see: ‘Strain of Speed: Mr Baldwin on Change in Industry’*Times* (14 April 1937): 14f. Also ‘Men and Machines’ *Times* (14 April 1937): 15c.

88. TNA MT13/17 London and Home Counties Traffic Advisory Committee 12th Annual Report 1936–1937 31,071 vehicles a day passing through Trafalgar Square in 1919 to 66,037 in 1935 T. See also *Court of inquiry, Day 1*, 11–12.

89. Fuller, *Radical Aristocrats,* 145.

90. See *Busman’s Punch*, Jan 1937.

91. 
*Industrial Courts Interim Report* Cmd. 5454, For the work of the industrial courts, see: I. G. Sharp, *Industrial Conciliation,* 347–360.

92. FD1/4084 Gastritis in Busmen III: M. Greenwood to E. Mellanby (11 May 1937): A. B. Hill to David Munro (23 May 1937).

93. Latour, *Never Been Modern*; Latour, ‘From Realpolitik to Dingpolitik, 14–41.

94. London Passenger Transport Act 1933 (1933 Chapter 14 23 and 24 Geo 5) sect. 88. This new object was questioned by Bevin who argued that the value of the B stock shares had been artificially inflated by the B stock shares.

95. Davies, ‘The London Bus Strike’. See also ‘Truths behind the Bus Strike’ *Reynolds News* (25 April 1937), 12.

96. Court of Inquiry, Day 1, 17.

97. Court of Inquiry, Day 1, 4. See also, 13.

98. 
*Court of Inquiry,* Day 1, 4, 5, 6. Over 900 traffic circulars giving new instructions on particular routes had been issued by the LPTB since 1933.

99. 
*Court of Inquiry,* Day 1, 6.

100. 
*Court of Inquiry* Day 1, 21.

101. 
*Court of Inquiry* Day 1, 7.

102. 
*Court of Inquiry,* Day 1, 9.

103. ibid., it is a problem that cannot be dealt with on the basis of statistics and averages and, 12: notes how the men ‘do not calculate life on averages, none of us does’ ’.

104. 
*Court of Inquiry,* Day 1, 34.

105. 
*Court of Inquiry* Day 1. 42.

106. 
*Court of Inquiry* Day 1*,* 37.

107. 
*Cmd.* 5464*,* 17, para., 27.

108. 
*Cmd.* 5464*,* 23, para 43; *London Evening Standard* Monday 3 May, 1a; [Editorial], ‘The Strike and the Board’, *The Spectator* (5 May 1937): 5.

109. [Ministry of Labour], *Health of London Central Busmen.*

110. On 8 Maybranches voted 47 to 3 against a return to work and 46 to 4 on 11 May. For figures on individual branch counts, see reports sent in by LPTB spotters held in London Transport Archives: LT0000401/002 Bus strike.

111. Clegg, *Labour Relations,* 126.

112. Glatter, ‘London Busmen’, 10; Clegg, *London Transport,* 127.

113. [Payne], *Defence of the Seven;* For a good overview see Fishman, *Communist Party,* 118–125.

114. Central Buses: New Agreement 15 June 1937 in Warwick MRC: MSS 26/TG/RES/P/1A.

115. Brown, ‘Breakaway busmen’s union’; Brown, ‘Busmen’s New Union’; Temple, ‘W. J. Brown’.

116. ‘Doctor indicts modern industry’, *Reynolds News* (9 May 1937): 10; [Macleans Brand Stomach Powder], ‘Stomach Trouble due to Speeding Up: A Remedy of National Importance’, *Reynolds News* (9 May 1937): 11; [Alka Seltzer], ‘How this London Bus Conductor overcame Indigestion’, *Derby Daily Telegraph* (15 October1937): 9; [Casell’s Tablets], ‘Overwork nearly cost him his job: Too tired to eat: too nervy to sleep’, *Reynolds News* (25 April 1937): 5. ‘Bus driver praises Yeast-Vite’ *Nottingham Evening Post* (10 September 1936): 10

117. ‘Busmen’s Wives Tell: The Heavy Toll of a Driver’s Job’, *Reynolds News* (2 May 1937), 5.

118. [Editorial], ‘Interest of Public Duty’, *Times* (7 May 1937): 17c. ‘Strike as Mutiny’, *Daily Sketch* (1 May 1937): 3.

119. ‘The Strike and the Board’, *The Spectator* (6 May 1937), 5.

120. The LPTB received 432 letters between 29 April and 31 May. While only 114 of these were explicitly anti-board, another 157 criticised or offered solutions to the culture of speed. TFL Archives: LT0000401/002.

121. Watson, ‘Busmen’; Chambers, *Unity Theatre,* 140–148;’Living Newspaper No. 1: Busmen’, *New Theatre No. 5* (March–April, 1938), 1.

122. Jardine, ‘Theatre’, 922–923.

123. ‘Living Newspaper No. 1’, V&A Theatre and Performance Archives THM/9/4/1/1.

124. Clark, *JBS Haldane.*

125. Haldane ‘Pain-Killers’, 155.

126. [Ministry of Labour], *Health of Central London Busmen,* para. 39.

127. Culpin, ‘Temperament and Digestive Disorders’.

128. Norman, ‘Medical Services’, 204–205. Norman, *Occupational Factors*; O’Dwyer, ‘Industrial Medical Officer’, 136; Doll and Jones, *Occupational Factors.*

## References

[CIT0001] Alexander Sally (2007). A New Civilization? London Surveyed 1928–1940s. *History Workshop Journal*.

[CIT0002] Alvarez W. C. (1929). Ways in Which Emotion Can Affect the Digestive Tract. *Journal of the American Medical Association*.

[CIT0003] Alvarez W. C. (1930). *Nervous Digestion*.

[CIT0004] Barford L. J. (1928). A Statistical Inquiry into the Aetiology, Symptoms, Signs and Results of Treatment in 166 Cases of Gastric and Duodenal Ulcer. *Guy’s Hospital Reports*.

[CIT0005] Barker T. C., Robbins Michael (1974). *A History of London Transport: Passenger Travel and the Development of the Metropolis: Volume II: The Twentieth Century to 1970*.

[CIT0006] Barman Christian (1979). *The Man Who Built London Transport: A Biography of Frank Pick*.

[CIT0007] Bennett Jane (2001). *The Enchantment of Modern Life: Attachments, Crossings, Ethics*.

[CIT0008] Berman Marshall (1988). *All that’s Solid Melts to Air: The Experience of Modernity*.

[CIT0009] Brown W. J. (1938). Why I Support the Breakaway Busmen’s Union. *Controversy: The Socialist Forum*.

[CIT0010] Brown W. J. (1938). The Busmen’s New Union: Why I am Lending a Hand. *RedTape*.

[CIT0011] Bullock Alan (1984). *The Life and Times of Ernest Bevin Vol. 1: Trade Union Leader, 1881–1940*.

[CIT0012] Cannon W. B. (1898). The Movements of the Stomach Studied by Means of the Rontgen Rays. *American Journal of Physiology*.

[CIT0013] Cannon W. B. (1907). The Influence of Emotional States on the Functions of the Alimentary Canal. *Journal of Medical Sciences*.

[CIT0014] Cannon W. B. (1911). *The Mechanical Factors of Digestion*.

[CIT0015] Cannon W. B. (1915). *Bodily Changes in Pain, Hunger, Fear and Rage*.

[CIT0016] Cannon W. B. (1937). *Digestion and Health*.

[CIT0017] Chambers Colin (1989). *The Story of the Unity Theatre*.

[CIT0018] Clegg Hugh (1950). *Labour Relations in London Transport*.

[CIT0019] Corfield Tony (1964). The History of the Union no. 29: Road Passenger Transport 8. *T & G. Record*.

[CIT0020] Cronin James (1989). The ‘Rank and File’ and the Social History of the Working Class. *International Review of Social History*.

[CIT0021] Cross Gary (1989). *The Quest for Time: The Reduction of Work in Britain and France, 1840–1940*.

[CIT0022] Csordas Thomas J. (1993). Somatic Modes of Attention. *Cultural Anthropology*.

[CIT0023] Culpin Millais (1929). Nervous Disease and Its Significance in Industry. *Medical Standard*.

[CIT0024] Culpin Millais (1930). The Need for Psychopathology. *The Lancet*.

[CIT0025] Culpin Millais (1930). *The Nervous Temperament*.

[CIT0026] Culpin Millais (1931). The Nervous Temperament: Its Assessment and Its Clinical Aspec. *British Journal of Medical Psychology*.

[CIT0027] Culpin Millais (1935). Temperament and Digestive Disorders. *BMJ*.

[CIT0028] Culpin Millais (1949). *Occupational Psychology*.

[CIT0029] Davies D. T. (1936). Some Observations on Peptic Ulcer. *The Lancet*.

[CIT0030] Davies D. T. (1937). Nervous Dyspepsia. *Practitioner*.

[CIT0031] Davies Ernest (1937). The London Bus Strike and the Public Corporation. *The Political Quarterly*.

[CIT0032] Davies D. T., Wilson A. T. M. (1937). *Lancet*.

[CIT0033] Departmental Committee on Compensation for Industrial Diseases (1933). *Second Report to the Right Honourable the Secretary of State for the Home Department by the Departmental Committee Appointed to Inquire and Report as to certain proceed extensions of the Schedule of Industrial disease to which Section 43 of the Workmen’s Compensation Act 1925, applies*.

[CIT0034] Derosières Alain (1998). *Politics of Large Numbers*.

[CIT0035] Doll Richard, Jones F. Avery (1951). *Occupational Factors in the Aetiology of Gastric and Duodenal Ulcers*.

[CIT0036] Dror Otniel (1988).

[CIT0037] Dunbar Flanders (1935). *Emotions and Bodily Changes*.

[CIT0038] Dunlop H. A. (1945). *Practitioner*.

[CIT0039] Eddington A. S. (1935). *New Pathways in Science*.

[CIT0040] Eisenstadt S. N. (2000). *Daedalus*.

[CIT0041] Faber Knud (1935). *Gastritis and its Consequences*.

[CIT0042] Farewell Vern, Johnson Tony, Armitage Peter (2006). *Statistics in Medicine*.

[CIT0043] Figlio Karl, Wright Peter, Treacher Andrew (1982). *The Problem of Medical Knowledge: Examining the Social Construction of Medicine*.

[CIT0044] Fishman Nina (1995). *The British Communist Party and the Trade Unions, 1933–1945*.

[CIT0045] Fuller Ken (1985). *Radical Aristocrats: London Busworkers from the 1890s to the 1980s*.

[CIT0046] German Lindsay, Rees John (2012). *A People’s History of London of London*.

[CIT0047] Giddens Anthony (1990). *Modernity and Self Identity*.

[CIT0048] Glatter Pete (1975). London Busmen: Rise and Fall of a Rank and File Movement. *International Socialism*.

[CIT0049] Greenwood M. (1935). *Epidemics and Crowd Diseases*.

[CIT0050] Guirdham Arthur Psychological Factors in Constipation. *Medical Press and Circular*.

[CIT0051] Hacking Ian, Douglas Mary, Hull David (1992). World-making by Kind-making: Child Abuse for Example. *How Classification Works: Nelson Goodman among the Social Sciences*.

[CIT0052] Hacking Ian, Sperber Dan, Premack David, Premack Ann J. (1994). The Looping Effects of Human Kinds. *Causal Cognition: A Multidisciplinary Approach*.

[CIT0053] Haldane J. B. S. (1941). *Science and Everyday Life*.

[CIT0054] Haldane J. S. (1931). Carbon Monoxide Poisoning with special relation to its Medico-Legal Aspects. *Transactions of the Medico-Legal Society*.

[CIT0055] Hardy Anne, Magnello Eileen, Morabia A. (2004). Statistical methods in epidemiology: Karl Pearson, Ronald Ross, Major Greenwood and Austin Bradford Hill, 1900–1945. *A History of Epidemiologic Methods and Concepts*.

[CIT0056] Harman N. (1937). Vision and the Motorist. *Practitioner*.

[CIT0057] Hawkins Herbert P. (1906). The Reality of Enterospasm. *BMJ*.

[CIT0058] Hayward Rhodri (2009). Enduring Emotions: James L. Halliday and the Invention of the Psychosocial. *Isis*.

[CIT0059] Hayward Rhodri, Richardson Angelique (2013a). Darwin’s Changing Expression and the Making of the Modern State. *After Darwin: Animals, Emotions and the Mind*.

[CIT0060] Hayward Rhodri, Taylor Barbara, Alexander Sally (2013b). Psychology and the Pursuit of Serenity in Post War Britain. *History and Psyche: Culture, Psychoanalysis and the Past*.

[CIT0061] Hibbs John (2004). *The History of British Bus Services*.

[CIT0062] Higgs E. (2000). Medical Statistics, Patronage and the State. *Medical History*.

[CIT0063] Hill Austin Bradford (1937). *An Investigation into the Sickness Experience among London Transport Workers with Special Reference to Digestive Disturbances*.

[CIT0064] Hill Austin Bradford (1937). *Principles of Medical Statistics*.

[CIT0065] Hinton Devon, Simon Naomi, Kirmayer L. J., Lemelson R., Cummings C. A. (2015). Cultural Neuroscience of Anxiety Disorders. *Revisioning Psychiatry: Cultural Phenomenology, Critical Neuroscience and Global Mental Health*.

[CIT0066] Hodson James Lansdale (1944). *Home Front. Being some account of journeys, meetings and what was said to me in and about England during 1942–1943*.

[CIT0067] Hurcomb C. (1945). Co-ordination of Transport: Great Britain 1935–44. *Journal of the Institute of Transport*.

[CIT0068] Hutchison Robert (1934). Hypochondriasis: Individual, Vicarious and Communal. *British Medical Journal*.

[CIT0069] Ihre Bengt (1938). *Human Gastric Secretion*.

[CIT0070] Jackson Mark (2014). *The Age of Stress*.

[CIT0071] Johnston James (1827). *An Essay on the Morbid Sensibility of the Stomach and Bowels etc*.

[CIT0072] Jones Edgar (2012). ‘The Gut War’: Functional Somatic Disorders in the UK During the Second World War. *History of the Human Sciences*.

[CIT0073] Jones Edgar, Jackson Mark (2015). Stomach for the Peace: Psychosomatic Disorders in UK Veterans and Civilians, 1945-55. *Stress in Post War Britain*.

[CIT0074] Jonsson F. A., Forth Christopher E., Carden Coyne Ana (2005). The Physiology of Hypochondria in Eighteenth-Century Britain. *Cultures of the Abdomen: Diet, Digestion and Fat in the Modern World*.

[CIT0075] Langdon-Brown Walter (1939). Visceral Symptoms in Emotional States. *Practitioner*.

[CIT0076] Latour Bruno (1998). *We Have Never Been Modern*.

[CIT0077] Latour Bruno, Latour Bruno, Webel Peter (2005). From Realpolitik to Dingpolitik—Or How to Make Things Publicings. *Making Things Public: Atmospheres of Democracy*.

[CIT0078] Leder Drew (1990). *The Absent Body*.

[CIT0079] Magnello E., Hardy A. (2002). *The Road to Medical Statistics*.

[CIT0080] Marriot John, O'Shea Alan, Nava Mica (1996). Sensation of the Abyss: The Urban Poor and Community. *Reflections on a Century of English Modernity*.

[CIT0081] Marx Karl https://www.marxists.org/archive/marx/works/1856/04/14.htm.

[CIT0082] Marx Karl, Engels Fredrich (1969). *Manifesto of the Communist Party*.

[CIT0083] Matthrews J. Rosser (1995). *Quantification and the Quest for Medical Certainty*.

[CIT0084] McCarrison Robert (1922). An Address on Faulty Food in Relation to Gastro-Intestinal Disorder. *The Lancet*.

[CIT0085] McIlroy John (2016). The Revival and Decline of Rank and File Movements in Britain During the 1930s. *Labor History*.

[CIT0086] Michael Mike (2000). *Reconnecting Culture, Technology and Nature*.

[CIT0087] Miller Ian (2010). The Mind and Stomach at War: Stress and Abdominal Illness in Britain c.1939–1945. *Medical History*.

[CIT0088] Miller Ian (2011). *A Modern History of the Stomach*.

[CIT0089] [Ministry of Labour] (1937). *Industrial Courts of Inquiry Act, 1919. Interim Report for a Court of Inquiry concerning the Stoppage of the London Central Omnibus Service*.

[CIT0090] [Ministry of Labour] (1939). *The Effect of Working Conditions upon the Health of London Central Busmen. Report of Conferences between representatives of the London Passenger Transport Board, the Transport and General Workers Union and the Medical Research Council under the Chairmanship of Sir John Forster*.

[CIT0091] Morrison Herbert (1933). *Socialisation and Transport: The Organisation of Socialised Industries with Particular Reference to the London Passenger Transport Bill*.

[CIT0092] Morton Hugh (1931). *Diseases of the Stomach*.

[CIT0093] Norman L. G. (1951). *Occupational Factors in the Aetiology of Gastric and Duodenal Ulcers*.

[CIT0094] Norman L. G., Mereweather E. R. A. (1954). Medical Services in Inland Transport. *Industrial Medicine and Hygiene*.

[CIT0095] O’Dwyer J. J, Ling T. M (1954). The Contribution of the Industrial Medical Officer. *Mental Health and Industrial Relations in Industry*.

[CIT0096] Owen Alex. M. Ling, Daunton Martin, Rieger Bernhard (2000). London: H. K. Le’lSelf in Fin-de-Siecle Britain.itain. *Meanings of Modernity: Britain from the Late Victorian Era to World War II*.

[CIT0097] Payne, William (1937). *London Busmen demand the right to live a little longer*.

[CIT0098] Payne, William (1937). *The Defence of the Seven Busmen: Being a summary fo the speeches made at the Inquiry held by the Transport and General Workers Union, June 22–30, 1937*.

[CIT0099] Pickering Andy, Scott Joan, Keates Deborah, Geertz Clifford (2001). Science as Alchemy. *Schools of Thought: Twenty-five Years of Interpretive Social Science*.

[CIT0100] Polanyi Karl (1946). *Origins of Our Time: The Great Transformation*.

[CIT0101] Price Richard (1989). ‘What’s in a Name?’ Workplace History and ‘Rank and Filism’. *International Review of Social History*.

[CIT0102] Rabinbach Anson (1990). *The Human Motor: Energy Fatigue and the Origins of Modernity*.

[CIT0103] Renshaw Geirge (1933). *The Story of the London Busmen’s Rank and File Movement: Why it was Formed; What has it Done; Its Future [The London Busmen’s Case no. 2]*.

[CIT0104] Saler Michael (1999). *The Avant Garde in Interwar England: Medieval Modernism and the London Underground*.

[CIT0105] Saler Michael (2002). Modernity and Enchantment: A Historiographic Review. *American Historical Review*.

[CIT0106] Schmidgen Henning (2014). *The Helmholtz Curves: Tracing Lost Time*.

[CIT0107] Shapin Stephen (2003). Trusting George Cheyne: Scientific Expertise, Common Sense, and Moral Authority in Early Eighteenth-century Dietetic Medicine. *Bulletin of the History of Medicine*.

[CIT0108] Sharp I. G. (1950). *Industrial Conciliation and Arbitration in Great Britain*.

[CIT0109] Smith Llewelyn (1934). *New Survey of London VIII*.

[CIT0110] Smith Martin The Return of the Rank and File. *International Socialism Journal*.

[CIT0111] Temple Richard (1999). W. J. Brown, Civil Service Unionism and London Busmen. *Historical Studies in Industrial Relations*.

[CIT0112] Thompson E. P. (1971). The Moral Economy of the English Crowd in the Eighteenth Century. *Past and Present*.

[CIT0113] Vernon James (2014). *Distant Strangers: How Britain Became Modern*.

[CIT0114] Walker Craig (2009). *On the Buses: The Complete Story*.

[CIT0115] Walton Jean (2010). Modernity and the Peristaltic Subject. *Neurology and Modernity: A Cultural History of Nervous Systems*.

[CIT0116] Watson Dom (1981). Busmen: Documentary and British Political Theatre in the 1930s. *Media Culture and Society*.

[CIT0117] Weber Max (1984). *Protestantism and the Spirit of Capitalism*.

[CIT0118] Weber Max, Gerth H. H., Wright Mills C. (1970). Science as a Vocation. *From Max Weber: Essays in Sociology*.

[CIT0119] Weller Peter (1993). *Ernest Bevin*.

[CIT0120] White Stephen K. (2000). *Sustaining Affirmation: The Strengths of Weak Ontology in Political Theory*.

[CIT0121] Williams Elizabeth (2010). Stomach and Psyche: Eating, Digestion, and Mental Illness in the Medicine of Philippe Pinel. *Bulletin of the History of Medicine*.

[CIT0122] Wittkower Eric (1935). Studies on the Influence of Emotions on the Functions of the Organs. *Journal of Mental Science*.

[CIT0123] Wrigley Chris, Wrigley Chris (1987). Trade Unions between the Wars. *A History of British Industrial Relations*.

[CIT0124] Young Allan (1995). *The Harmony of Illusions: Inventing Post-traumatic Stress Disorder*.

[CIT0125] Young Allan, Lawrence Christopher, Weisz George (1998). Walter Cannon and the Psychophysiology of Fear. *Greater than the Parts: Medical Holism in Biomedicine, 1920–1950*.

[CIT0126] Zeitlin Jonathan (1989). ‘Rank and Filism’ in British Labour History: A Critique. *International Review of Social History*.

